# Autophagy dysregulation by mutant fused in sarcoma—implications for amyotrophic lateral sclerosis

**DOI:** 10.1038/cddis.2015.311

**Published:** 2015-10-29

**Authors:** K Y Soo, J D Atkin

**Affiliations:** 1 Department of Biochemistry and Genetics, La Trobe Institute for Molecular Science, La Trobe University, Bundoora, Victoria, Australia; 2 Department of Biomedical Sciences, Faculty of Medicine and Health Sciences, Macquarie University, North Ryde, New South Wales, Australia

Neurodegenerative diseases such as amyotrophic lateral sclerosis (ALS) are associated with disturbances of proteostasis, consistent with accumulation of insoluble protein aggregates as a major pathological hallmark. Autophagy, a protein degradation process that delivers cellular constituents to the lysosome, is an important component of proteostasis. Increasing evidence implies that autophagy dysfunction is important in the pathogenesis of ALS. Previous studies have demonstrated the accumulation of autophagosomes in spinal cords of sporadic ALS (sALS) patients,^1^ and mutant proteins central to familial ALS (fALS), Tar DNA-binding protein 43 (TDP-43) and superoxide dismutase 1 (SOD1), also dysregulate autophagy. Furthermore, mutations in proteins that function in autophagy also cause fALS, implying that autophagy has an important role in disease pathology. Fused in sarcoma/translocated in liposarcoma (FUS) is another protein integrally involved in ALS, which bears structural and functional similarities to TDP-43, and at least 50 mutations in the *FUS* gene cause 4% of fALS cases. A recent study published in *Cell Death Discovery*, demonstrates that ALS-associated mutant FUS also dysregulates the early stages of autophagy,^2^ by inhibiting the formation of the autophagosome, an early stage in the process. Furthermore, this report reveals that overexpression of Rab1, which mediates autophagosome biogenesis, can rescue these autophagy defects, thus identifying a new possible therapeutic target for ALS.

ALS is a fatal, rapidly progressing disorder, which targets upper and lower motor neurons in the brain, brainstem and spinal cord, and there is currently no effective treatment. Most cases of ALS (90%) are sporadic but 10% of cases are familial and primarily linked to mutations in genes encoding C9ORF72, SOD1, TDP-43, FUS, amongst others. Both FUS and TDP-43 are RNA-binding proteins that mislocalize from the nucleus to the cytoplasm in ALS. Misfolded TDP-43 forms misfolded intracellular inclusions within motor neurons in almost all cases (97%) of ALS. Misfolded FUS is present in the inclusions of fALS patients bearing FUS mutations, and misfolded wild-type (WT) FUS has also been detected in sALS inclusions.

The formation of misfolded protein aggregates in ALS suggests that there is an imbalance between misfolded protein degradation and generation. Although the ubiquitin-proteosome system (UPS) mainly degrades short-lived proteins, autophagy removes long-lived or misfolded proteins. Autophagy begins by the formation of the omegasome from the endoplasmic reticulum (ER), which subsequently forms the autophagosome membrane. The autophagosome then fuses with the lysosome, where its contents are degraded. Although subtypes of autophagy exist, involving different mechanisms of substrate delivery, lysosomal degradation is the common feature. Autophagy is highly dependent on efficient intracellular trafficking processes. Rab GTPases regulate all membrane trafficking events and specific Rab proteins mediate autophagy-related trafficking, in particular Rab1, Rab5, Rab7 and Rab11.

The presence of misfolded protein inclusions suggests that autophagy is inefficient or dysfunctional in ALS and there is increasing evidence for this notion. The levels of autophagic marker LC3-II are increased in spinal cords of SOD1^G93A^^3^ and SOD1^H46R^ mice.^4^ Administration of autophagy inducer trehalose enhanced SOD1 degradation by autophagy in NSC-34 neuronal cells.^5^ TDP-43 is degraded by both the UPS and autophagy^6^ and autophagy induction by rapamycin^7^ or trehalose reduces TDP-43 aggregates.^8^ Furthermore, induction of autophagy enhances survival in cells expressing mutant TDP-43.^9^ TDP-43 aggregates co-localize with LC3 and p62^6^ and overexpression of p62 reduces TDP-43 aggregation.^10^ Interestingly, mutations in autophagy proteins p62, TBK1, optineurin and ubiquilin-2 also cause fALS, and the normal cellular function of C9ORF72, which is linked to most cases of fALS, is related to autophagy.^11^ However it remains unknown if mutant FUS dysregulates autophagy in ALS.

Soo *et al.*^2^ now demonstrate that two ALS-associated mutant FUS proteins, P525L and R522G, inhibit autophagy in neuronal cells. Autophagosome formation, investigated by quantifying LC3-II expression using immunoblotting and immunocytochemistry, was impaired in cells expressing mutant FUS compared with control cells expressing WT FUS or untransfected cells. Similarly, the accumulation of ubiquitinated proteins, revealed by increased levels of autophagy substrates p62, Htt mutant and NBR1, and inhibition of omegasome formation, assessed using specific marker DFCP1, was also detected in mutant FUS expressing cells. Furthermore, Soo *et al.*^2^ also demonstrated that autolysosome formation is inhibited in cells expressing mutant FUS, consistent with the formation of fewer autophagosomes in these cells (Figure 1a). These findings were subsequently confirmed using primary cortical neurons. Hence, together these findings imply that mutant FUS inhibits the early stages of autophagy, by inhibiting omegasome and hence autophagosome formation (Figure 1a). Evidence of autophagy dysregulation was also detected in motor neurons from a fALS patient bearing the R521C FUS mutation. In contrast to the findings obtained in cell culture, autophagosomes were found to accumulate in fALS patient tissues. However, the cellular and human findings are not difficult to reconcile. As mutant FUS inhibits autophagosome formation, it is possible that by disease end stage in human patient tissues, functional autophagy is inhibited, leading to autophagosome accumulation (Figure 1b). Alternatively, the non-canonical autophagy pathway may be induced in human spinal cords (Figure 1b). Non-canonical autophagy is triggered by the accumulation of reactive oxygen species, including those generated from dysfunctional autophagy. Hence it can bypass conventional autophagy and the formation of the omegasome.

Soo *et al.*^2^ also demonstrate that overexpression of Rab1, which is involved in autophagosome formation, restores the autophagy defects induced by mutant FUS. Stress granules (SGs) are also linked to aggregation and pathogenesis in ALS, and ALS-associated mutant FUS proteins R495X, R514S and R521G incorporate abnormally into SGs, in contrast to WT FUS, which is not recruited to SGs under the same conditions.^12, 13^ SGs form *in vitro* under conditions of oxidative stress, ER stress or heat shock, in iPSCs obtained from ALS patients with FUS mutations, and *in vivo* in zebrafish models.^12, 13^ ALS-associated mutant FUS also interferes with SG assembly and morphology, suggesting that mutant FUS impairs the stress response.^14^ Soo *et al.*^2^ also demonstrate that restoration of autophagy by Rab1 prevents mutant FUS recruitment into SGs, and reduces the size of SGs formed, thus providing a link between Rab1, autophagy and SGs. These data are consistent with another recent study,^15^ in which restoration of autophagy by rapamycin reduced the recruitment of FUS into SGs. Hence, together these studies suggest that Rab1 has a protective role in neurodegeneration in ALS.

In conclusion, autophagy has emerged as an important field in recent years and is now well documented in ALS and other neurodegenerative diseases. However, the contribution of autophagy to the pathology of ALS remains unclear, and previous studies involving TDP-43 and SOD1 have yielded conflicting findings. The study by Soo *et al.*^2^ demonstrates that autophagy is dysregulated by mutant FUS, another important protein in ALS. Modulation of autophagy by Rab1 and other means may have potential as a novel and effective therapeutic target in ALS. This is an important first step for the design of useful therapies for this devastating disease.

## Figures and Tables

**Figure 1:**
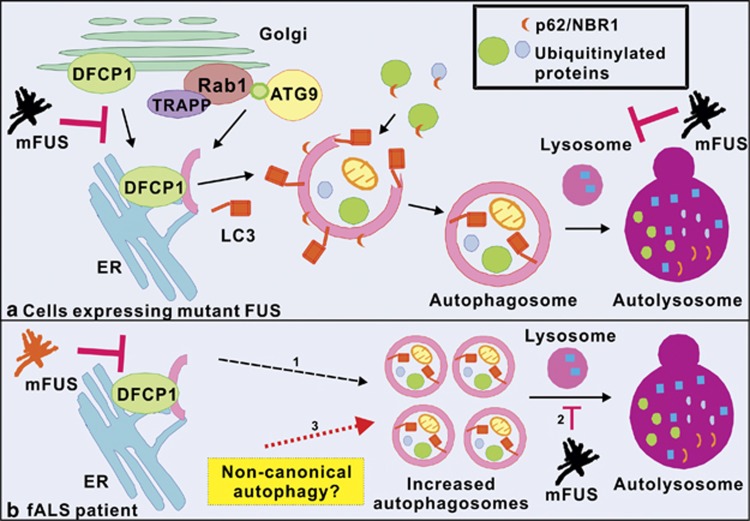
Autophagy dysfunction in mutant FUS-linked ALS (**a**) Initiation of autophagy occurs at the ER. Firstly, an omegasome is formed from the ER membrane. The omegasome is characterised by the presence of DFCP1, which is mobilized from the Golgi apparatus. The isolation membrane then forms the omegasome. Additional membrane obtained from Golgi vesicles, containing membrane associated proteins ATG9, TRAPP as well as Rab1, is added to the isolation membrane to form the double layered autophagosome. LC3, a widely used autophagosome marker, is then recruited to the autophagosome membrane. Autophagic receptors p62 and NBR1, which both bind to ubiquitinated proteins and target ubiquitinated protein to autophagosomes, are also recruited to the autophagosome membrane. The autophagosome then fuses with the lysosome to form the autolysosome. In cells expressing mutant FUS, mutant FUS inhibits the formation of the autophagosome, omegasome, and autolysosome. (**b**) In fALS patients, (1) although autophagosomes do not form as readily in mutant FUS expressing cells, over the course of human disease, eventually functional autophagy is inhibited, leading to the accumulation of autophagosomes (dashed arrow). (2) The lack of autolysosomes may lead to an increase the number of autophagosomes in human patient motor neurons. (3) Another possibility is that the increase in LC3 vesicles in fALS patient motor neurons is due to induction of the non-canonical autophagy pathway (red dotted arrow). mFUS, mutant FUS
